# siRNA-Mediated Silencing of CIP2A Enhances Docetaxel Activity Against PC-3 Prostate Cancer Cells

**DOI:** 10.15171/apb.2017.076

**Published:** 2017-12-31

**Authors:** Saiedeh Razi Soofiyani, Akbar Mohammad Hoseini, Ali Mohammadi, Vahid Khaze Shahgoli, Behzad Baradaran, Mohammad Saeid Hejazi

**Affiliations:** ^1^Immunology Research Center, Tabriz University of Medical Sciences, Tabriz, Iran.; ^2^Department of Molecular Medicine, Faculty of Advanced Biomedical Sciences, Tabriz University of Medical Sciences, Tabriz, Iran.; ^3^Molecular Medicine Research Center, Tabriz University of Medical Sciences, Tabriz, Iran.

**Keywords:** CIP2A, Docetaxel, Prostate cancer, siRNA

## Abstract

***Purpose:*** Cancerous inhibitor of protein phosphatase 2A (CIP2A) is an identified human oncoprotein which modulates malignant cell growth. It is overexpressed in human prostate cancer and in most of the human malignancies. The aim of this study was to investigate the effects of CIP2A silencing on the sensitivity of PC-3 prostate cancer cells to docetaxel chemotherapy.

***Methods:*** PC-3 cells were transfected using CIP2A siRNA. CIP2A mRNA and protein expression were assessed after CIP2A gene silencing using q-RT PCR and Western blotting. Proliferation and apoptosis were analyzed after treatment with docetaxol using MTT assay, DAPI staining, and flow cytometry, respectively.

***Results:*** Silencing of CIP2A enhanced the sensitivity of PC-3 cells to docetaxel by strengthening docetaxel induced cell growth inhibition and apoptosis against PC-3 cells.

***Conclusion:*** Silencing of CIP2A may potentiate the cytotoxic effects of docetaxel and this might be a promising therapeutic approach in prostate cancer treatment.

## Introduction

Prostate cancer is the common malignancy among men and it accounts for the second cause of cancer-related death in men.^1,2^ In spite of significant efforts in the treatment of prostate cancer, conventional therapies could not successfully treat the tumors. Therefore, most of the patients will develop castration resistant prostate cancer (CRPC) for the duration of 18-24 months and this is associated with poor prognosis.^3,4^ Chemotherapy, using docetaxel as a taxan family member, is the first choice for the treatment of CRPC.^5,6^ The anti-tumor effects of docetaxel depend on its ability to promote microtubules polymerization and stabilization, which leads to cell cycle arrest and apoptosis.^7,8^ The toxicity and undesirable events in docetaxel based treatment reduce the therapeutic efficacy and limit its tolerated dose.^9^ Lack of efficient treatments shows the importance of additional means to develop effective therapeutic approaches in the treatment of prostate cancer. One of the feasible approaches consists of combining a chemotherapy agent with silencing specific proteins involved in proliferation and survival of the cancer cells using RNA interference technology due to RNAi's high specificity, noticeable efficacy, and low toxicity when compared with the other reverse genetic technologies.^10-12^ Cancerous inhibitor of protein phosphatase 2A (CIP2A), a human oncoprotein, is generally overexpressed in most human malignancies and its overexpression is closely associated with poor outcome in patients.^13,14^ It promotes malignant cell growth and tumor progression.^15^ It is overexpressed in prostate cancer samples and cell lines.^16,17^

Since, the effect of CIP2A suppression on docetaxel induced cytotoxicity against prostate cancer cells has not been reported, this study investigated the effects of CIP2A silencing on the sensitivity of PC-3 cells to docetaxel chemotherapy.

## Materials and Methods

### Cell culture and drug

PC-3, human prostate cancer cells (Pasteur Institute, Iran), were cultured in RPMI-1640 medium (GIBCO, Carlsbad, CA, USA) supplemented with 10% FBS (Invitrogen Life Technologies), containing 100 U/mL penicillin and 100 μg/mL streptomycin (Sigma-Aldrich, St. Louis, MO, USA) in a humidified atmosphere of 5% CO_2_ at 37°C. Docetaxel, 10 mg/ml (Hospira UK Co., Ltd) was used at the concentrations of 0.39 to 50 nM.

## siRNA transfection

CIP2A siRNA, negative control siRNA (not homologous to any gene), and siRNA transfection reagent were purchased from Santa Cruz Biotechnology, USA. CIP2A siRNA (human) is a pool of 3 different siRNA duplexes (Table 1). To knock down the CIP2A expression with CIP2A siRNA, the cells at 2×10^5^/well density were seeded in 6-well plate and cultured overnight before transfection using CIP2A siRNA according to the manufacturer’s instruction. Briefly, siRNA transfection medium was used to dilute siRNAs and siRNA transfection reagent. The diluted solutions were mixed gently and incubated at room temperature for 15 to 30 min. Then, the complexes were added to each well and were incubated in a humidified CO_2_ incubator at 37°C for 6 h. 1 ml of RPMI-1640 medium containing 20% FBS was added to each well. After 24, 48, and 72 h, down-regulation of CIP2A was monitored using quantitative RT-PCR (qRT-PCR) and Western blotting.

**Table 1:** Three different siRNA duplexes.

**siRNA duplexs pool**	**Sense**	**Antisense**
sc-77964A	5′ CUAGCAGUAGACAUUGAAAtt 3′	5′ UUUCAAUGUCUACUGCUAGtt 3′
sc-77964B	5′ GUACCACUCUUAUAGAACAtt 3′	5′ UGUUCUAUAAGAGUGGUACtt 3′
sc-77964C	5′ GGAAGUAAGCUUCUACAAAtt 3′	5′ UUUGUAGAAGCUUACUUCCtt 3′

### Quantitative RT-PCR

Total RNA was extracted from the cells using the RiboEx total RNA extraction kit (GeneAll Biotechnology CO, LTD, Korea). cDNA was synthesized using random Hexamer (rH) primer and Moloney murine leukemia virus (M-MuLV) reverse transcriptase (Fermentas Life Sciences, Vilnius, Lithuania). The primer sequences were as follows: GAPDH, forward 5´ CAAGATCATCACCAATGCCT 3’; reverse: 5´ CCCATCACGCCACAGTTTCC 3’; CIP2A, forward 5´GATTATTGGCAAATCTTTGTCGG 3’; reverse 5´CTGATGAATGTTTCGAGCATGG 3’. qPCR was done using SYBR Green Master Mix (Ampliqon III, VWR-Bie Berntsen, Denmark) on LightCycler® 96 System (Roche Diagnostics, Mannheim, Germany) as follows: 95°C for 10 min, followed by 40 cycles at 94°C for 10 s, 60°C for 30 s, and 72°C for 20 s, using GAPDH as an endogenous control for sample normalization. The relative expression level of mRNAs was calculated using the 2 -^ΔΔCt)^ method (Livak and Schmittgen, 2001).

### Western blotting analysis

The cells lysate was prepared using RIPA Lysis Buffer System (Santa Cruz Biotechnology, USA) according to the manufacturer’s instruction and was quantified using NanoDrop (Thermo Scientific, Wilmington, USA). 50 µL of protein was separated using 10% SDS-PAGE gel electrophoresis and transferred to polyvinyl difluoride membranes (Roche Diagnostics GmbH). After blocking using blocking buffer containing 0.5% Tween-20 for 2 h at 25°C, the membranes were incubated with primary antibodies against CIP2A (1:200; Santa Cruz Biotechnology, USA) and monoclonal antibody against β-actin (1:5000, Abcam) overnight at 4°C. After washing, the membranes were incubated using the secondary antibodies conjugated to horseradish peroxidase (1:3000, Razi Biotech Co, Tehran, Iran) for 2 h at room temperature. Protein bands were visualized using enhanced BM chemiluminescence blotting substrate POD (Roche Diagnostics GmbH, Mannheim, Germany) and Western blot imaging system (Sabz BIOMEDICALS, Iran). To quantify the bands intensity, ImageJ software version 1.44 software (National Institutes of Health, Bethesda, USA) was used.

### Cytotoxicity assay

PC-3 cells (7×10^3^) were seeded into 96-well plates. The cells were transfected using CIP2A siRNA. 48 h after CIP2A-siRNA transfection, the cells were treated using different concentrations of the docetaxol (0.39-50 nM) for 24 h. Then, 50 μL of MTT solution (2 mg/mL) was added to each well and was incubated in the humidified incubator containing 5% CO_2_ at 37°C for 4 h. Then, 200 μl of DMSO and 25 μl Sorenson buffer were added to each well. The optical density (OD) of each well was measured using a microplate reader (Awareness Technology, Palm City, FL, USA) at a wavelength of 570 nm. IC_50_, the concentration which induced 50% cytotoxicity, was calculated using GraphPad Prism 6.01 software (GraphPad Software Inc., San Diego, CA, USA).

### Treatment and control groups 

PC-3 cells were subdivided into 5 groups: (A) Control group, un-treated PC-3 cells; (B) Negative control (NC) siRNA treated group, scrambled siRNA-transfected PC-3 cells; (C) CIP2A siRNA treated group, PC-3 cells were transfected using 80 pmol CIP2A siRNA; (D) DTX group, PC-3 cells were exposed to IC_50_ docetaxel treatment (3.6 nM); and (E) CIP2A siRNA + DTX group, PC-3 cells were pre-treated using CIP2A siRNA and exposed to IC_50_ docetaxel (3.6 nM).

### Combination effect analysis

To study the interaction between CIP2A siRNA and docetaxel, combination effect analysis was performed based on Chou and Talalay principles.^18^ CDI, the coefficient of drug interaction value, was determined using the formula:

CDI = SAB / (SA × SB).

Where SAB is the survival rate of the combination group relative to the control group, SA and SB are the survival rate of docetaxel and CIP2A siRNA relative to the control group, respectively. CDI<1, CDI=1 and CDI>1 indicate synergistic, additive, and antagonistic effects, respectively.

### DAPI staining

PC-3 cells were seeded at 7×10^3^ cells/well density in 96-well plates. Following the aforementioned treatments, the cells were fixed with 4% paraformaldehyde for 60 min at 37°C, washed three times with PBS, permeablized with 0.1% Triton-X-100 for 10 min, washed with PBS, and incubated with 50 μL of DAPI solution (1/2000 dilution, in 1x TBST buffer; Sigma, St. Louis, MO, USA) for 10 min in the dark. After washing with PBS, the DAPI signals were measured using Cytation 5 (Biotk) at the wavelength of 377 to 477 nm. The apoptotic cells were characterized by condensed chromatin and fragmented nuclei.

### Apoptosis assay by Annexin-V FITC/PI staining 

Forty eight hours after CIP2A siRNA transfection, the cells were exposed to 3.6 nM of docetaxel for 24 h. After the exposure, the cells were harvested and resuspended in cold PBS for analysis. Annexin-V FITC/PI staining kit (Roche Diagnostics, Germany) was used to stain the cells for additional analysis of cell death according to the manufacturer's instructions. Data were collected using a BD FACS Calibur flow cytometer (San Jose, CA, USA) and were analyzed with Flowing software.

### Statistical analysis 

All data were presented as mean ± standard deviation (SD). Statistical differences between groups were analyzed using T test and Two-way analysis of variance (ANOVA) followed by Dunnett’s multiple comparisons with GraphPad Prism software, La Jolla California USA, http://www.graphpad.com. P value less than 0.05 was considered statistically significant.

## Results and Discussion

### CIP2A siRNA down-regulated CIP2A expression in PC-3 cells

**Figure 1:**
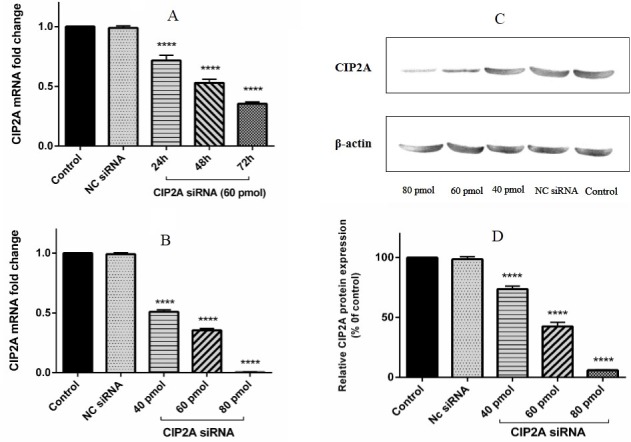
**Suppression of CIP2A expression at mRNA and protein levels by CIP2A siRNA in PC-3 Cells.** (A) q-RT PCR analysis of CIP2A expression levels in PC-3 cells following transfection with 60 mol of CIP2A specific siRNA or NC siRNA for 24, 48, and 72 h. (B) CIP2A mRNA expression levels in PC-3 cells transfected with 40, 60, and 80 pmol of specific siRNA for 72 h (1B). CIP2A expression was down regulated in time and dose dependent manner in the cells treated with CIP2A siRNA. (C) The CIP2A protein expression was determined by western blotting following transfection of cells with 40, 60 and 80 pmol of CIP2A siRNA or NC siRNA. The results showed a remarkable reduction in CIP2A expression after CIP2A transfection with 80 pmol of CIP2A siRNA, when compared with the protein level in the control group. (D) The intensity of bands was quantified and normalized to the β-actin (as an internal control) relative to CIP2A expression in the control group. The results were shown as mean±SD (n=3); ****P < 0.0001 versus control.

To investigate the effect of CIP2A siRNA on CIP2A expression in PC-3 cells, the CIP2A expression in PC-3 cells was knocked down by specific CIP2A siRNA for 24, 48, and 72 h with 60 pmol of CIP2A siRNA. The CIP2A expression level was analyzed using qRT-PCR. The relative expression of CIP2A mRNA for 24, 48 and 72 h after transfection were 0.71, 0.52 and 0.34, respectively (Figure 1A). Then, to setup the optimum dose of CIP2A siRNA, the cells were transfected using 40, 60, and 80 pmol of siRNA for 72 h. qRT-PCR and Western blot analysis were used to determine the expression of CIP2A at mRNA and protein levels following transfection using different doses. The relative CIP2A mRNA expression level for doses 40, 60, and 80 pmol of CIP2A siRNA was 0.50, 0.35, and 0.01, while the relative CIP2A protein expression levels were %73, %42, and 5% of the control, respectively. The results showed that CIP2A expression was remarkably reduced in cells transfected using 80 pmol of CIP2A siRNA as compared to the control. The optimum knockdown dose and time were achieved on 80 pmol of siRNA, 72 h post transfection (Figure 1B, 1C and 1D). Accordingly, the following tests were performed using the same condition.

No significant differences were found in CIP2A mRNA and protein levels in the control groups (un-treated cells and negative siRNA group; p > 0.05).

Therefore, the results showed that silencing CIP2A with specific siRNA significantly reduced the expression of CIP2A in a time and dose dependent manner.

### siRNA mediated CIP2A down-regulation inhibited the proliferation of PC-3 cells 

**Figure 2:**
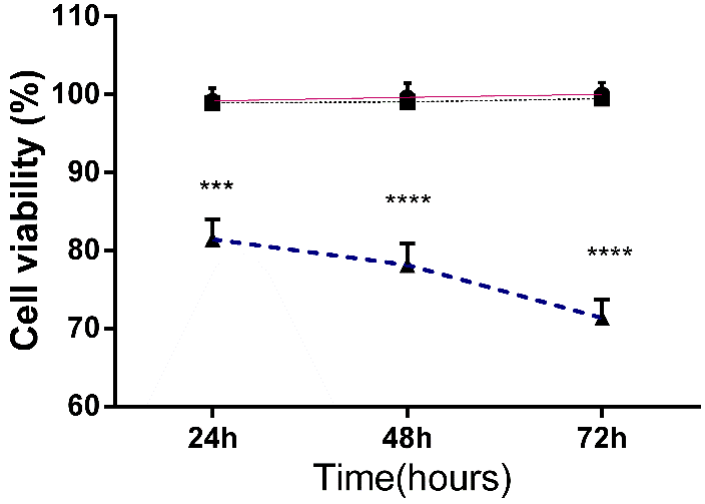
**Effect of CIP2A silencing on PC-3 cells viability.** PC-3 cells transfected with CIP2A siRNA or NC siRNA for 24, 48 and 72 h, then the effect of treatment on cell viability was determined by MTT assay. The results were shown as mean±SD (n=3); **P<0.01, ***P<0.001, ****P<0.0001 versus control. ●,■ and▲ indicated to control group, NC siRNA treated group and CIP2A siRNA treated group , respectively.

In order to analyze the effect of CIP2A down-regulation on the proliferation and viability of PC-3 cells, MTT assay was used at different time points after CIP2A siRNA transfection. As shown in Figure 2, compared to the control group, CIP2A siRNA significantly decreased the proliferation and cell viability of P-C3 cells at 24, 48 and 72 h after transfection in a time dependent manner (p<0.0001). 24 h after CIP2A siRNA transfection, the cell viability was reduced to 81% and later dropped to 70% at 72 h. Also, no significant differences in cell viability were observed between the NC siRNA transfected cells and the un-treated control group (p>0.05). Therefore, CIP2A plays a key role in the proliferation of prostate cancer cells.

### CIP2A siRNA synergistically enhanced the cytotoxic effects of docetaxel

**Figure 3:**
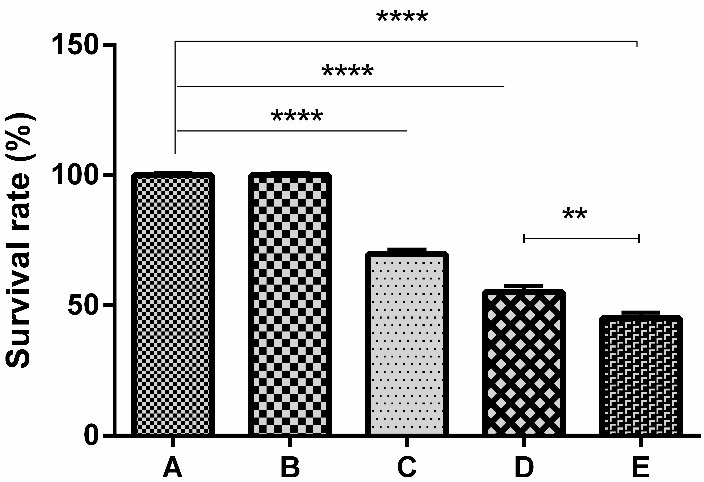
**Effect of CIP2A siRNA on docetaxel induced cytotoxicity against PC-3 cells.** PC-3 cells were transfected with CIP2A siRNA or exposed to 3.6 nM of docetaxel alone and a combination of them as shown in the methods section. The survival rate was analyzed using MTT assay. The results were shown as mean±SD (n=3); ****P<0.0001. A, B, C, D, and E indicated in the studied groups were as stated in the methods section.

**Figure 4:**
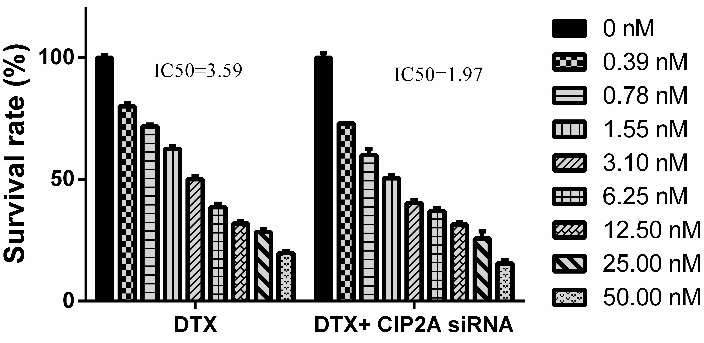
**Effect of CIP2A silencing on the chemosensitivity of the PC-3 cells to docetaxel.** 48 h after transfection with CIP2A siRNA, the cells were exposed to 0.39 to 50 nM of docetaxel for 24 h. Then, the treatments cytotoxicity was determined using MTT assay. The results were shown as mean±SD (n=3); **P<0.01 versus docetaxel treated alone cells.

Sequential treatment regime was designed to explore whether CIP2A silencing could enhance the chemosensitivity of PC-3 cells to docetaxel. PC-3 cells were pre-treated with CIP2A siRNA for 48 h followed by 0.39 to 50 nM of docetaxel for 24 h and the effects of the mentioned treatment were evaluated using MTT assay. As shown in Figure 3, CIP2A siRNA significantly decreased the cell survival rate to 70% and the combination of docetaxel and CIP2A siRNA significantly decreased the cell survival rate to 45% as compared to the control. The IC_50_ value of docetaxel was reduced from 3.59 to 1.97 nM after CIP2A siRNA transfection (Figures 3 and 4). Also, the CDI values were less than 1 in all the concentrations of docetaxel which pointed to the synergistic effect between CIP2A siRNA and docetaxel (Table 2). Thus, it is suggested that CIP2A down-regulation could sensitize PC-3 cells to docetaxel.

**Table 2:** The CDI value for combination of CIP2A siRNA and 0.39 to 50 nM of docetaxel.

Concentration of CIP2A siRNA and docetaxel	CDI
80 pmol+0.39 nM	0.73
80 pmol+0.78 nM	0.68
80 pmol+1.55 nM	0.65
80 pmol+3.1 nM	0.68
80 pmol+6.25 nM	0.77
80 pmol+12.5 nM	0.86
80 pmol+25 nM	0.71
80 pmol+50 nM	0.70

## Silencing of CIP2A increased apoptosis induced by docetaxel

**Figure 5:**
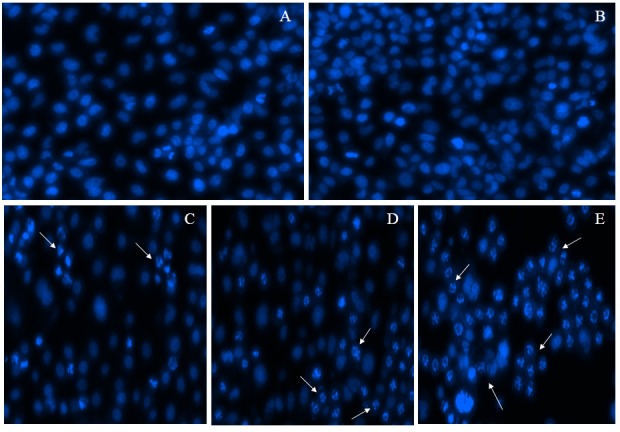
**Nuclear morphological changes of PC-3 cells by DAPI staining.** The white arrow marks indicate condensed and damaged nuclear material. A, B, C, D, and E indicated in the studied groups were as mentioned in the methods section.

To determine whether CIP2A suppression could enhance docetaxel induced apoptosis, DAPI and Annexin-V FITC/PI staining were used. Distinctive apoptosis-related morphological changes such as nuclear condensation were observed and nuclear fragmentation in the cells was treated using CIP2A siRNA, docetaxel, and both of them on the florescence micrographs when compared with NC siRNA treated and un-treated control groups (Figure 5). These changes, such as nuclear condensation and fragmentation, are in line with the apoptosis. The apoptotic effect of CIP2A siRNA and docetaxel on PC-3 cells was further confirmed by Annexin-V/PI staining.

**Figure 6:**
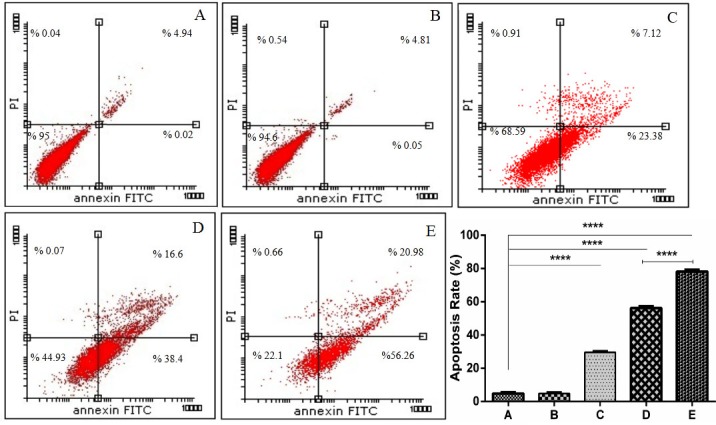
**Effect of CIP2A silencing on docetaxel induced apoptosis.** PC-3 cells were treated with CIP2A siRNA and docetaxel alone or in combination as mentioned in methods section. The apoptosis was analyzed using AnnexinV/PI staining. The results are expressed as mean±SD (n=3); ****P<0.0001 versus control. A, B, C, D, and E indicated in the studied groups were as mentioned in the methods section.

Figure 6 shows that *the apoptosis percentage* in PC-3 cells treated with CIP2A siRNA + 3.6 nM docetaxel was significantly higher than CIP2A siRNA treated cells and docetaxel treated cells alone (p<0.0001). However, no significant change was observed for NC siRNA treated group in the apoptosis rate when compared with the un-treated cells. Therefore, the knockdown of CIP2A enhanced the apoptosis induction effect by docetaxel on PC-3 cells. Fang et al. showed that down-regulation of CIP2A enhanced the paclitaxel induced apoptosis in human ovarian cancer cells.^19^ Also, Zhang et al. showed that the depletion of CIP2A sensitized ovarian cancer cells to cisplatin.^20^ These findings are fully in agreement with the results of similar studies, which further verify the important role of CIP2A in the survival and proliferation of cancer cells.

## Conclusion

Conclusively, the present study showed that silencing of CIP2A could enhance the antitumor effects of docetaxel against PC-3 cells. Therefore, targeting of CIP2A can be considered as a novel strategy for targeted prostate cancer synergy therapy.

## Acknowledgments

The authors would like to thank the Immunology Research Center (IRC), Tabriz University of Medical Sciences, for the financial support of this project (No. 94/59).

## Ethical Issues

Not applicable

## Conflict of Interest

The authors declare no conflict of interests.
